# Abdominal aortic thrombosis and tuberculosis: an uncommon association

**DOI:** 10.1093/gastro/gou021

**Published:** 2014-04-12

**Authors:** Alka Sharma, Vishal Sharma

**Affiliations:** ^1^Department of Medicine, Government Medical College and Hospital, Sector 32, Chandigarh, India and ^2^Department of Gastroenterology, Postgraduate Institute of Medical Education and Research, Chandigarh, India

**Keywords:** Vasculitis, thrombosis, tuberculosis, aorta

## Abstract

Thrombosis of the abdominal aorta is an uncommon event and usually occurs in a diseased vessel. We report a case of a 42-year-old male who presented with abdominal distension and was found to have tuberculosis-related ascites and was incidentally found to have aortic thrombosis. The patient improved with four-drug anti-tubercular therapy and anticoagulation. The occurrence of non-occlusive thrombosis of the abdominal aorta in tuberculosis is unusual.

## INTRODUCTION

Aortic thrombosis is uncommon and may result in embolic or ischaemic manifestations. Abdominal aortic thrombosis usually occurs in the setting of underlying atherosclerosis or aortitis, as in syphilis or aneurysms; it has occasionally been reported to be due to pelvic peritonitis [1]. In cases where thrombosis occurs in the absence of underlying aortic disease, hypercoagulable conditions must be sought for. Abdominal tuberculosis has not been previously reported to cause aortic thrombosis.

## CASE REPORT

**Figure 1.**
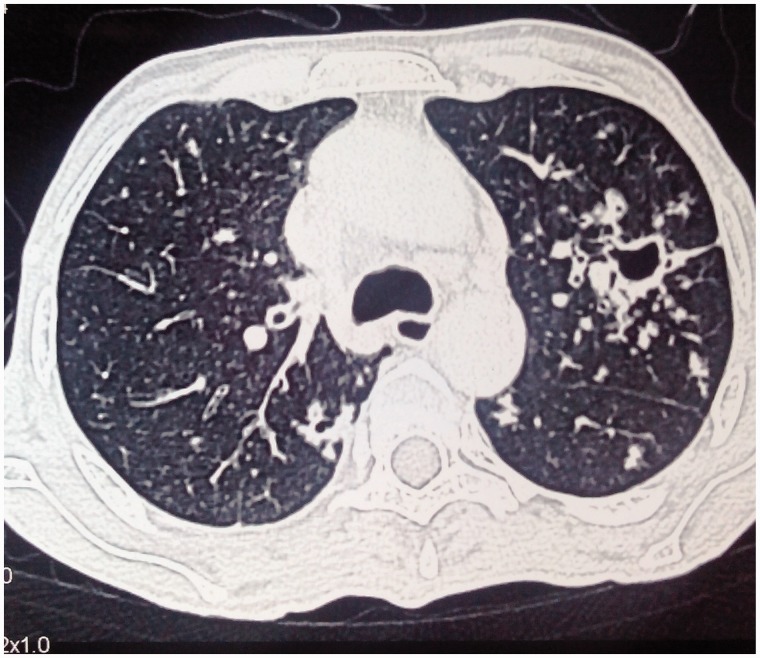
Changes of bronchiectasis in the lungs.

**Figure 2.**
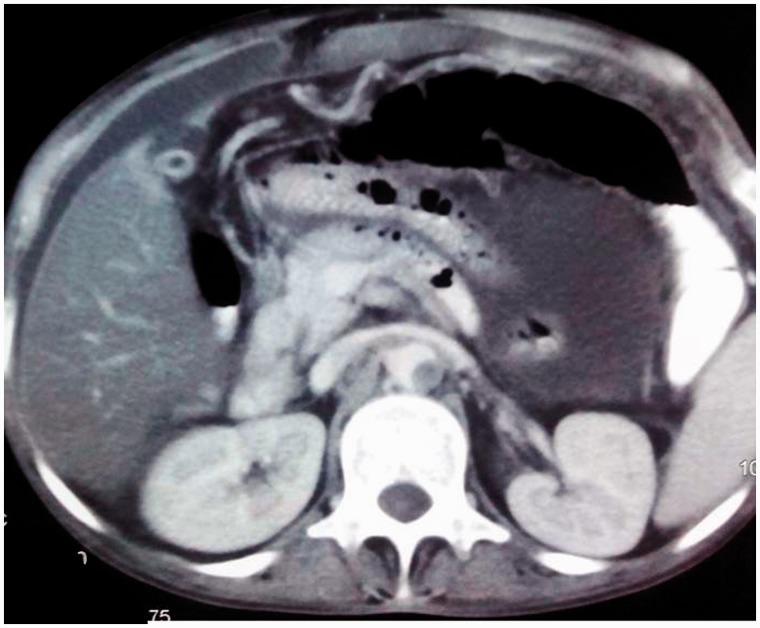
Computed tomography image of abdomen showing ascites, peritoneal enhancement and aortic thrombosis.

A 42-year-old male was admitted with a history of abdominal distension, abdominal pain and fever of two months duration. The abdominal distension was insidious in onset and slowly progressive and was associated with loss of appetite and fever. The fever was low-grade and not associated with chills or rigors, but was associated with night sweats. The patient had multiple matted cervical lymph nodes. On examination, his abdomen was distended and shifting dullness was elicited. Fine needle aspiration cytology from a cervical lymph node yielded caseous material showing granulomatous inflammation and stain testing for acid-fast bacilli was positive. Ultrasound scanning of the abdomen confirmed the presence of ascites. An ascitic tap was carried out and a straw-coloured fluid with protein count of 2.9 g/dL and a serum-ascites albumin gradient (SAAG) of 0.5 g/dL was obtained. Cytology revealed 800 cells, predominantly lymphocytes, and fluid adenosine deaminase (ADA) value was 65 IU/mL. The patient's abdominal and chest computed tomography (CT) revealed evidence of bronchiectatic changes in the lung (Figure 1) and ascites with peritoneal enhancement. Incidentally, the abdominal aorta showed evidence of incomplete occlusion due to a thrombus (Figure 2). The patient had no symptoms of arterial compromise or claudication. The patient was started on four-drug, weight-based, anti-tubercular therapy (isoniazid, rifampin, pyrazinamide and ethambutol) and low molecular weight heparin and warfarin. He improved, with reduction in abdominal distension. Repeat CT imaging at 3 months confirmed the resolution of ascites and the aortic thrombus. Anticoagulation with warfarin was continued for 6 months and stopped thereafter. The patient was later evaluated for an underlying hypercoagulable state including Protein C and S, antithrombin III, Leiden V mutation, antiphospholipid syndrome and hyperhomocysteinemia but the work-up was non-contributory.

## DISCUSSION

Aortic thrombosis is an uncommon event, due to high blood flow rates in the aorta. The presentation is usually in the form of acute abdominal or limb or visceral ischaemia. In patients with occlusive aortic thrombosis, timely intervention is warranted lest the outcome be adverse. Chronic non-occlusive aortic thrombosis is, however, more common and occurs with underlying aortic disease such as aneurysm, or related to hypercoagulable states [2]. Such patients can be treated with anticoagulation—as in the present case. Although both venous and arterial thrombosis has been reported with active tuberculosis, the association of disseminated tuberculosis and aortic thrombosis is rare and has not been previously documented [3, 4]. In a report of 30 cases of arterial thrombosis, six had underlying active tuberculosi; however none had peritoneal tuberculosis [3]. Although aortic thrombosis usually involves the infrarenal aorta, in our patient the suprarenal abdominal aorta was involved. The patient had peritoneal tuberculosis, as witnessed by the presence of ascites, peritoneal enhancement with high protein and low SAAG ascites with elevated ADA levels. It is unclear as to what should predispose patients with tuberculosis to develop aortic thrombosis. Although vasculitis in the cerebral and retinal vasculature is recognised, it is not clear if the infectious process also affects the aorta to provide a milieu conducive to thrombosis. We report this case, as it shows a rare complication of peritoneal tuberculosis.

**Conflict of interest:** none declared.
